# Biomimetic Human Disease Model of SARS‐CoV‐2‐Induced Lung Injury and Immune Responses on Organ Chip System

**DOI:** 10.1002/advs.202002928

**Published:** 2020-12-21

**Authors:** Min Zhang, Peng Wang, Ronghua Luo, Yaqing Wang, Zhongyu Li, Yaqiong Guo, Yulin Yao, Minghua Li, Tingting Tao, Wenwen Chen, Jianbao Han, Haitao Liu, Kangli Cui, Xu Zhang, Yongtang Zheng, Jianhua Qin

**Affiliations:** ^1^ Division of Biotechnology CAS Key Laboratory of SSAC Dalian Institute of Chemical Physics Chinese Academy of Sciences Dalian 116023 China; ^2^ University of Chinese Academy of Sciences Beijing 100049 China; ^3^ Kunming National High‐Level Bio‐safety Research Center for Non‐human Primates Center for Biosafety Mega‐Science Kunming Institute of Zoology Chinese Academy of Sciences Kunming 650107 China; ^4^ Key Laboratory of Animal Models and Human Disease Mechanisms of Chinese Academy of Sciences and Yunnan Province Kunming Institute of Zoology Chinese Academy of Sciences Kunming Yunnan 650223 China; ^5^ KIZ‐CUHK Joint Laboratory of Bioresources and Molecular Research in Common Diseases Kunming Institute of Zoology Chinese Academy of Sciences Kunming Yunnan 650223 China; ^6^ Kunming College of Life Science University of Chinese Academy of Sciences Kunming Yunnan 650204 China; ^7^ Institute for Stem Cell and Regeneration Chinese Academy of Sciences Beijing 100101 China; ^8^ CAS Center for Excellence in Brain Science and Intelligence Technology Chinese Academy of Sciences Shanghai 200031 China

**Keywords:** COVID‐19, disease model, drug testing, organ chip, SARS‐CoV‐2

## Abstract

Coronavirus disease 2019 (COVID‐19) is a global pandemic caused by severe acute respiratory syndrome coronavirus 2 (SARS‐CoV‐2). The models that can accurately resemble human‐relevant responses to viral infection are lacking. Here, a biomimetic human disease model on chip that allows to recapitulate lung injury and immune responses induced by SARS‐CoV‐2 in vitro at organ level is created. This human alveolar chip reproduce the key features of alveolar‐capillary barrier by coculture of human alveolar epithelium, microvascular endothelium, and circulating immune cells under fluidic flow in normal and disease. Upon SARS‐CoV‐2 infection, the epithelium exhibits higher susceptibility to virus than endothelium. Transcriptional analyses show activated innate immune responses in epithelium and cytokine‐dependent pathways in endothelium at day 3 post‐infection, revealing the distinctive responses in different cell types. Notably, viral infection causes the immune cell recruitment, endothelium detachment, and increased inflammatory cytokines release, suggesting the crucial role of immune cells involved in alveolar barrier injury and exacerbated inflammation. Treatment with remdesivir can inhibit viral replication and alleviate barrier disruption on chip. This organ chip model can closely mirror human‐relevant responses to SARS‐CoV‐2 infection, which is difficult to be achieved by in vitro models, providing a unique platform for COVID‐19 research and drug development.

## Graphical abstract

Coronavirus disease 2019 (COVID‐19) is a global pandemic caused by severe acute respiratory syndrome coronavirus 2 (SARS‐CoV‐2). Here, a biomimetic human alveolar infection model is built using organ chip that allows to recapitulate the lung injury and immune responses induced by SARS‐CoV‐2 at organ level. It provides a unique and rapid platform to accelerate COVID‐19 research and drug development.

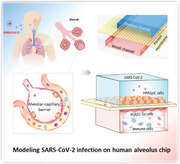

## 1 Introduction

Coronavirus disease 2019 (COVID‐19) pandemic broke out in late 2019 and quickly became a global epidemic.^[^ ^1^, ^2^, ^3^, ^4^ ^]^ Severe acute respiratory syndrome coronavirus 2 (SARS‐CoV‐2), the causative virus of COVID‐19, has infected over ten million individuals by July 2020 according to the report from World Health Organization (WHO), and the number of patients and deaths continues to increase globally. COVID‐19 patients exhibit multiple clinical features including fever, dry cough, and ground‐glass opacity.^[^ ^5^, ^6^ ^]^ The human lung is the primary target of SARS‐CoV‐2 infection, which is characterized by the process ranging from mild syndrome to severe lung injury, eventually multi‐organ failure. Many severe COVID‐19 cases develop into progressive respiratory failure, resulting in death due to diffuse alveolar damage, inflammation, and pneumonia.^[^ ^5^, ^7^, ^8^, ^9^ ^]^ Based on pathological features of COVID‐19 by biopsy samples, inflammatory infiltration of mononuclear cells or lymphocytes can be observed in lung interstitial tissues or alveolar cavities.^[^ ^9^, ^10^ ^]^ Particularly, previous studies from severe patients have suggested that the excessive inflammatory cytokine storm induced by SARS‐CoV‐2 often results in aberrant immunopathology and lethal outcome. However, the in‐depth mechanism of the pathogenesis of COVID‐19 remains unclear.

At present, the research of SARS‐CoV‐2 infection mostly relies on monolayer cultures of cell lines or primary tissue cells,^[^ ^11^, ^12^ ^]^ human organoids,^[^ ^13^, ^14^ ^]^ and animal models.^[^ ^15^, ^16^ ^]^ However, all of these models have their limitations. For example, monolayer cell cultures are oversimplified and cannot exhibit the complex structure and functions of human organ‐specific microenvironments in vivo. Human organoids (e.g., lung organoids) can provide multiple cell types and more complex tissue‐specific functions, but they cannot simulate organ level features of lung, such as tissue–tissue interfaces, blood flow, cross‐talk between epithelium and endothelium, and immune cell–host responses, which are essential for the pathological progression of viral infections in pulmonary tissues in vivo. In addition, animal models of SARS‐CoV‐2 infection have been established to validate the treatment of drugs or vaccines.^[^ ^15^, ^16^ ^]^ However, given the species difference, their targeted organs or systemic responses to SARS‐CoV‐2 infection may be significantly different from that of human individuals. Moreover, expensive costs, time‐consuming processes, and animal ethics should be carefully considered. As such, it is highly desirable to develop alternative preclinical models to better reflect the pathophysiology of human organs to accelerate SARS‐CoV‐2 research and the development of COVID‐19 candidate therapeutics.

Significant advances in bioengineered organs‐on‐chips technology have made it possible to reconstruct 3D human organotypic models in vitro by recapitulating the key functions of living organisms in microfluidic culture devices, such as intestine, heart, liver, and lung.^[^ ^17^, ^18^, ^19^, ^20^, ^21^ ^]^ They can resemble the organ physiology in a human‐relevant manner, holding great potentials in disease studies, drug testing, and virology.^[^ ^17^, ^22^, ^23^, ^24^, ^25^ ^]^ Inberg's group successfully proposed the engineered lung‐on‐chips in a multilayered microfluidic device that enables to reconstitute the air–liquid interfaces^[^ ^17^ ^]^ and applied them to study respiratory diseases, such as pulmonary edema,^[^ ^26^ ^]^ asthma, and toxicity assessment.^[^ ^27^ ^]^ Other types of lung chip models were developed to reproduce alveolar‐capillary interfaces with flexible configurations to assess the environmental pollutants induced toxicity.^[^ ^24^, ^28^ ^]^ More recently, lung chip was also attempted to study the study the host–virus interactions by influenza virus and pseudotyped SARS‐CoV‐2, and screen approved drugs as potential therapeutics.^[^ ^23^, ^29^ ^]^ Junaid et al. reported the modeling of Ebola hemorrhagic syndrome in a microvessel‐on‐a‐chip system to test the effect of candidate drugs.^[^ ^30^ ^]^

**Figure 1:**
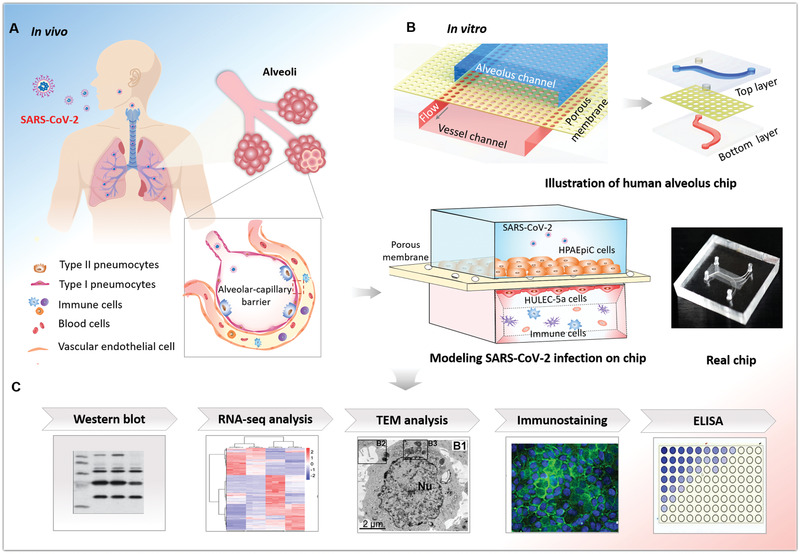
Schematic diagram of microengineered human alveolus chip infected by SARS‐CoV‐2. A) Illustration of 3D human alveolar‐capillary barrier in vivo. B) The configuration of biomimetic human alveolus chip infected by SARS‐CoV‐2. The device consists of upper alveolar epithelial channel (blue) and lower pulmonary microvascular endothelial channel (red) separated by a porous PDMS membrane. The alveolar‐capillary interface is formed by coculture of alveolar epithelial cells (HPAEpiC) and pulmonary microvascular endothelial cells (HULEC‐5a) on chip under fluid flow conditions. The established alveolus chip is exposed to SARS‐CoV2 on the epithelial layer. Human immune cells are infused into the bottom vascular channel during the progression of virus infection. C) The responses of human alveolus chip to SARS‐CoV‐2 are analyzed using different methods.

According to clinical findings, lung is the primary target organ in the progress of COVID‐19. In vivo, alveolus is the core functional unit of lung, in which the alveolar‐capillary barrier plays a vital role in maintaining gas exchange and preventing the invasion of external hazardous substances or virus infection (**Figure** **1**A). This functional barrier is mainly composed of human alveolar epithelial and vascular endothelial cells that interact across a 3D extracellular matrix (ECM). More clinical evidences indicate that SARS‐CoV‐2 infection can damage alveolar function in severe patients. In order to create the human lung infection model by SARS‐CoV‐2 in vitro, we initially designed and constructed a biomimetic human alveolus chip in a multilayer microfluidic culture device under physiological fluid flow conditions. The lung alveolar chip consisted of two perfused channels (alveolar lumen and vascular channels) sandwiched with an ECM‐coated porous membrane that enables the coculture of human alveolus epithelial cells, pulmonary microvascular endothelial cells, and immune cells under perfused media flow. The device can reproduce the critical features of the human alveolar‐capillary barrier by synergistically combining human epithelium–endothelium interactions, 3D ECM, and mechanical fluid cues. Upon SARS‐CoV‐2 infection, we systematically analyzed the response of distinct cell types to the virus through immunostaining and RNA‐sequencing (RNA‐seq) analysis. By adding circulating immune cells in the vascular channel, we examined the pathological changes of epithelium–endothelium interface and inflammatory responses after virus infection, as well as the potential antiviral therapeutics. This human disease model on chip offers a robust microsystem for studying the human responses to infectious viruses, providing a unique platform for accelerating COVID‐19 research and development of effective therapeutics.

## 2 Results

### 2.1. Characterization of Microengineered Human Alveolus Chip

The purpose of this work is to create a human disease model of SARS‐CoV‐2 infection and study human responses in vitro using microengineered lung chip device. Initially, we designed and fabricated the microfluidic lung chip, which consists of two channels separated by a thin and porous polydimethylsiloxane (PDMS) membrane coated with ECM (Figure 1B). The ECM‐coated PDMS membrane allows the coculture of different cell types on the opposite sides of the membrane, mimicking the tissue interface in vivo. Moreover, the membrane with many pores (5 µm in diameter) distribution‐is beneficial to substance diffusion and the interactions between the upper and lower cell layers (Figure S1, Supporting Information). The culture chamber permits the perfusion of media flow, which can promote nutrients exchange and waste removal.

Human alveolar epithelial type II cell (AT II) line (HPAEpiC) and lung microvasculature cell line (HULEC‐5a) were seeded on the upper and lower sides of the porous membrane, respectively. These two types of cells were cultured for 3 d until confluent into monolayers under continuous media flow (50 µL h^−1^) in upper and bottom channels, thus forming the alveolus epithelium–endothelium tissue interface. The integrity of the formed tissue barrier was assessed by the expression of adherent junction proteins in both human epithelium and endothelium. Immunostaining analysis showed that epithelial cells can form adherent junctions identified by E‐cadherin, and endothelial cells formed conjunctions identified by VE‐cadherin, respectively (Figure S2, Supporting Information). Furthermore, the integrity of barrier under different culture conditions was assessed by the diffusion rate of FITC‐dextran between the two parallel channels (Figure S3, Supporting Information). The barrier permeability under fluid flow is lower than that in static cultures, indicating the important role of flow in maintaining the function and integrity of the alveolar‐capillary barrier. These results suggest that the established human alveolus chip could effectively mimic the physiological alveolar‐capillary barrier.

### 2.1 SARS‐CoV‐2 Infection in Human Alveolus Chip

**Figure 2:**
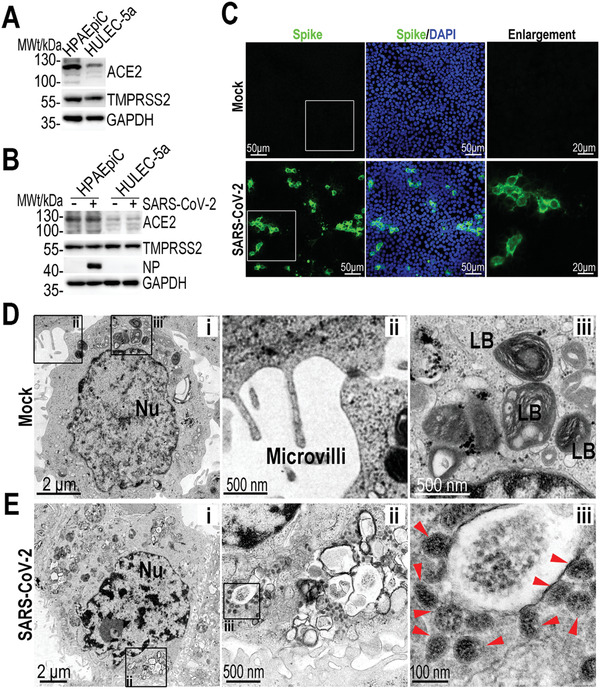
Examination of SARS‐CoV‐2 infection in HPAEpiC cells. A) Analysis of ACE2 and TMPRSS2 proteins expression levels in HPAEpiC and HULEC‐5a cells by western blot. The results are representative blot from three experiments. GAPDH is served as a loading control. B) Analysis of ACE2, TMPRSS2, and viral nucleoprotein (NP) expression levels in mock‐ or SARS‐CoV‐2‐infected cells at day 3 post‐infection. The results are representative blot from three independent experiments. C) Immunofluorescent images showed the viral Spike protein (Spike protein S1 subunit of SARS‐CoV‐2) staining in HPAEpiC cells at day 3 post‐infection. C) TEM images of mock HPAEpiC cells without virus infection. i) The overall image of HPAEpiC cell. ii) The enlarged image of microvilli on free surface of HPAEpiC cell. iii) The enlarged image of lamellar bodies (LB) within cell body. D) TEM images of SRAS‐CoV‐2‐infected HPAEpiC cells. i) The overall image of the infected cell. ii) Clusters of viruses within cell body. iii) Enlarged image of virus particles. Three independent experiments were performed (*n* = 3).

It has been reported that SARS‐CoV‐2 uses ACE2 as a host receptor for cellular entry, and transmembrane serine proteinase 2 (TMPRSS2) for Spike protein priming.^[^ ^12^, ^31^, ^32^, ^33^ ^]^ Prior to creating the SARS‐CoV‐2 infection model based on human alveolus chip, we sought to identify the susceptibility of alveolar epithelial cells to this virus. Initially, we examined the expression of ACE2 and TMPRSS2 proteins in HPAEpiC and HULEC‐5a cells, respectively (**Figure** **2**A). The western blot data showed the positive expression of ACE2 and TMPRSS2 in both cell types,^[^ ^12^, ^34^ ^]^ and the expression of ACE2 in HPAEpiC cells was higher than that in HULEC‐5a cells. To further determine the expression of ACE2 and TMPRSS2 proteins in HPAEpiC cells and HULEC‐5a cells after viral infection, we infected these two cell types separately in monolayer cultures at a multiplicity of infection (MOI) of 10. At day 3 post‐induction, the protein expressions were analyzed by western blot. The results showed a higher expression of viral NP protein in HPAEpiC cells as compared to that observed in HULEC‐5a cells, indicating the higher susceptibility to SARS‐CoV‐2 infection in alveolar epithelial cells than pulmonary microvascular endothelial cells. Moreover, there are no obvious changes in the expression levels of ACE2 and TMPRSS2 in both cell types after viral infection (Figure 2B), suggesting the less effects of viral infection on the protein levels of ACE2 and TMPRSS2 in host cells. Similarly, upon SARS‐CoV‐2 infection, no significant difference was observed in the protein levels of these two proteins between the HPAEpiC cells cultured alone and cocultures with HULEC‐5a cells (Figure S4, Supporting Information). The results suggest the expressions of ACE2 and TMPRSS2 proteins in HPAEpiC cells maintains stable, which are not dependent on viral infection and culture conditions. HPAEpiC were then infected with SARS‐CoV‐2 at a MOI of 10, and detected by immunofluorescent staining to check the infection efficiency. At day 3 post‐infection, more than 20% Spike protein‐positive cells were observed (Figure 2C). To further examine the ultrastructure of SARS‐CoV‐2‐infected cells, transmission electron microscope (TEM) analysis of mock‐ or SARS‐CoV‐2‐infected HPAEpiC cells was carried out (Figure 2D,E). The TEM images showed that mock cells exhibited primary AT II cell‐like morphological characteristics, including small cellular size with square or round shape (Figure 2Di), microvilli on free surface (Figure 2Dii), and lamellar bodies within cell body (Figure 2Diii).^[^ ^35^, ^36^ ^]^ In the infected cells, lots of viral particles were detected and distributed in clusters within cell bodies as shown in Figure 2E, indicating the susceptibility of HPAEpiC cells to SARS‐CoV‐2 infection.

**Figure 3:**
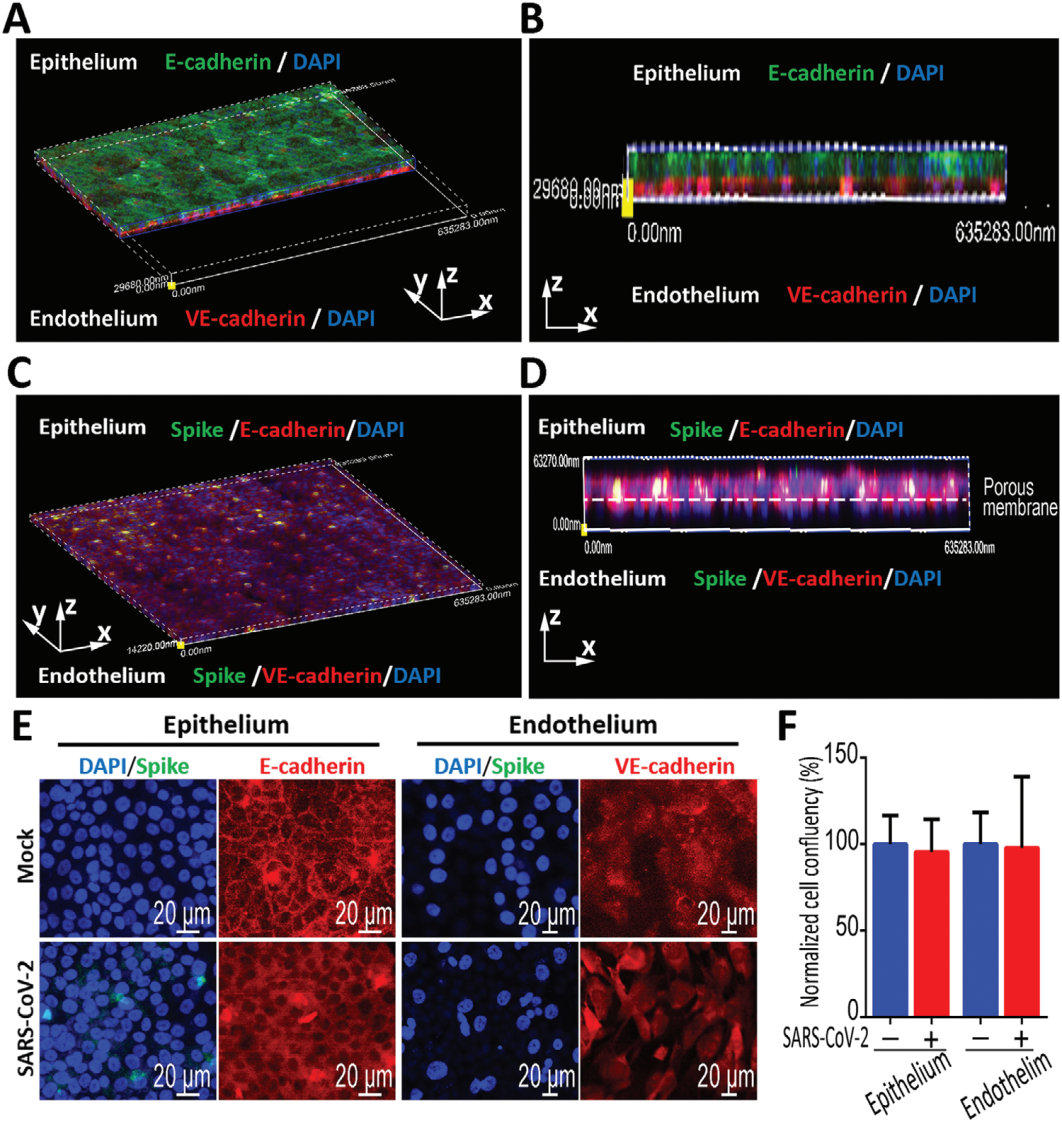
Characterization of infection and replication of SARS‐CoV‐2 in human alveolus chip. A) 3D reconstructed confocal image of alveolar epithelium (E‐cadherin) and pulmonary microvascular endothelium (VE‐cadherin) in the alveolus chip. B) Side view of alveolar epithelium (E‐cadherin) and pulmonary microvascular endothelium (VE‐cadherin). C) 3D reconstructed confocal image of human alveolar‐capillary‐barrier at day 3 post‐infection. D) Side view of the formed alveolar barrier after viral infection. SARS‐CoV‐2 infection was predominantly identified in epithelium layer by viral Spike protein expression. E) Confocal immunofluorescent microscopy images showed the effects of SARS‐CoV‐2 infection (Spike protein) on the human epithelium (E‐cadherin) and endothelium (VE‐cadherin) of chip at day 3 post‐infection. F) Cell confluency of epithelium and endothelium on chip was examined with or without SARS‐CoV‐2 infection. Data were presented as mean ± SD. Three chips were quantified for each group.

To mimic alveolar infection by SARS‐CoV‐2, the virus was inoculated into the upper alveolus channel of the chip at a MOI of 10, and the cells were cultured for 3 d. In the mock‐infected chip, the HPAEpiC cells and HULEC‐5a cells formed confluent cell layers on both sides of the porous membrane (Figure 3A,B). While in the infected‐chip, the predominated expression of Spike protein was observed in epithelial cells, demonstrating viral infection and massive replication in alveolar epithelium (Figure 3C,D), but not in endothelial cells. In addition, there are no significant changes in the organization of adherent junction proteins in HPAEpiC cells (E‐cadherin) and HULEC‐5a cells (VE‐cadherin), as well as the confluent rate of epithelial cells and endothelial cells (Figure 3E,F). These results suggested that SARS‐CoV‐2 can primarily infect and replicate in the alveolar epithelial cells, but not in endothelial cells.

### 2.2 Transcriptional Analysis of Host Cells Responses to SARS‐CoV‐2 Infection

**Figure 4:**
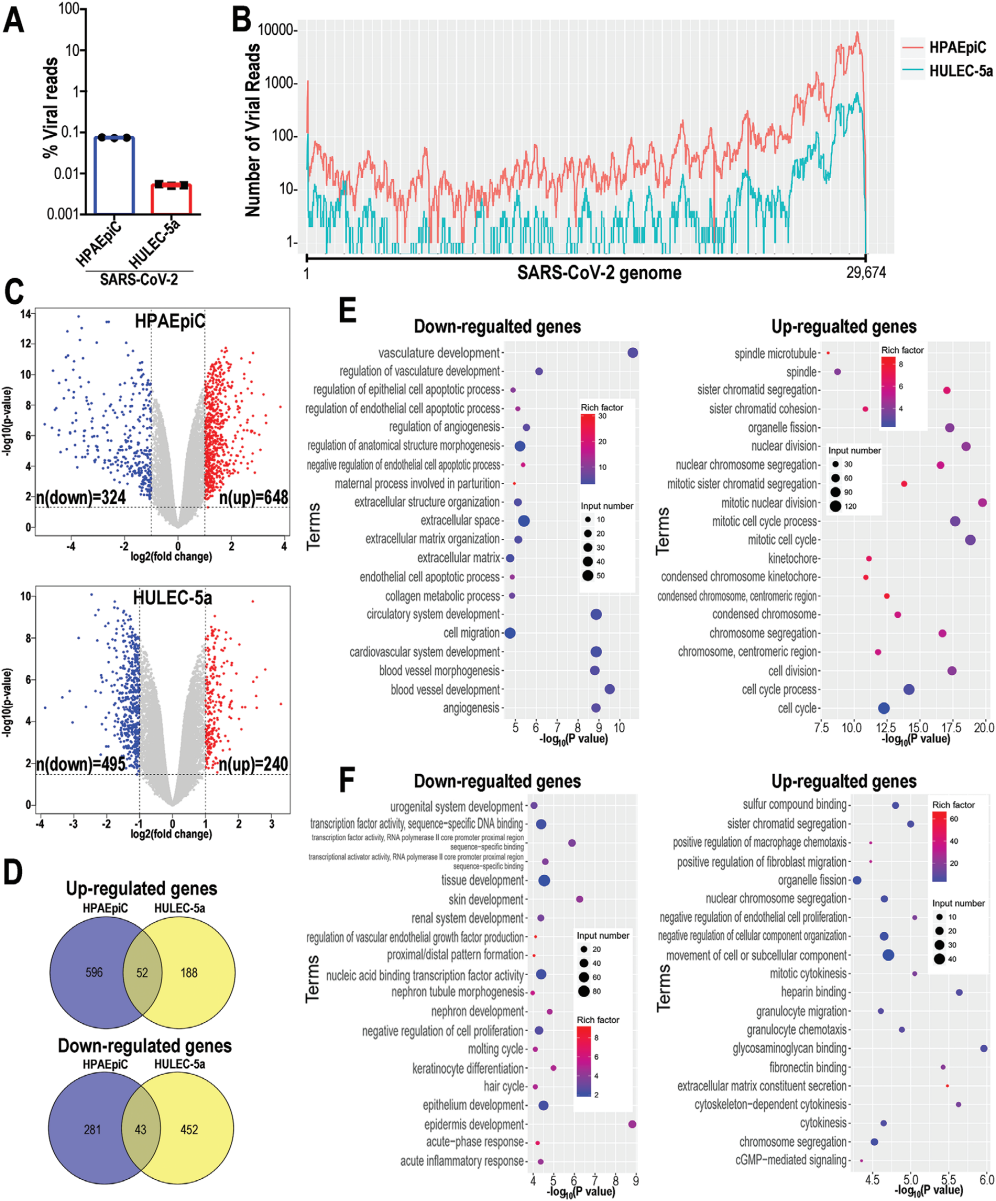
Transcriptional analysis of HPAEpiC cells and HULEC‐5a cells to SARS‐CoV‐2 infection in human alveolus chip. A) Viral replication levels in HPAEpiC and HULEC‐5a cells. The ratio of virus‐aligned reads over total reads is indicated for the viral replication level for each sample. Three independent experiments were performed (*n* = 3). B) Read coverage of viral reads along the SARS‐CoV‐2 genome for the infected‐HPAEpiC cells or HULEC‐5a cells. The graph indicated the viral reads number per position of the viral genome in the infected‐HPAEpiC cells or HULEC‐5a cells. This graph is representative of three independent experiments. C) Volcano plots showing the regulated genes of cells following SARS‐CoV‐2 infection. Genes differentially expressed with fold change over 2.0 and *p* < 0.05 were marked in color. *P* values were calculated using a two‐sided, unpaired Student's *t*‐test with equal variance assumed (*n* = 3 independent biological samples). D) Venn diagrams depicting the differentially expressed genes (DEGs) shared or unique between each comparison. E) GO enrichment analysis of DEGs in HPAEpiC cells following SARS‐CoV‐2 infection. F) GO enrichment analysis of DEGs in HULEC‐5a cells following SARS‐CoV‐2 infection. E,F) The color of the dots represents the rich factor and the size represents the input number for each GO term. The horizontal axis indicates the significance of enrichment (−log10 (*P* value)), and the vertical axis indicates the 20 most enriched GO terms.

In order to fully understand the transcriptional responses to SARS‐CoV‐2 infection, we performed RNA‐seq analysis of HPAEpiC and HULEC‐5a cells following viral infection in the alveolus chip. Briefly, 3 d after infection, HPAEpiC cells and HULEC‐5a cells were collected separately and analyzed by RNA sequencing. First, the ratio of virus‐aligned reads over total reads in each sample was calculated to estimate the viral replication levels in these two cell types. The results showed that the ratio of viral reads in HPAEpiC cells is much higher than HULEC‐5a cells (**Figure** **4**A,B), which are consistent with the western blot (Figure 2B) and immunostaining analysis (Figure 3D). It revealed that human alveolar epithelial cells were more permissive to SARS‐CoV‐2 infection than microvascular endothelial cells, similar to the histopathological findings from autopsy reports.^[^ ^37^ ^]^

Volcano plots showed that SARS‐CoV‐2 infection induced global transcriptome modulations in both HPAEpiC cells and HPAEpiC cells (Figure 4C). To identify the differentially expressed genes (DEGs) in the two cell types, the cutoff values for the fold change and *P* value were set to 2.0 and 0.05, respectively. Among the DEGs, 972 genes (324 down‐regulated genes and 648 up‐regulated genes) were significantly modulated in HPAEpiC cells, while 735 genes (495 down‐regulated genes and 240 up‐regulated genes) were significantly modulated in HULEC‐5a cells. By combining the two data sets, we found the two cell types only shared 52 overlapping upregulated DEGs (6.2% of total up‐regulated DEGs) and only 43 overlapping downregulated DEGs (5.5% of total down‐regulated DEGs) (Figure 4D). These results suggested that the alveolar epithelial and pulmonary microvascular endothelial cells showed distinct transcriptional responses to SARS‐CoV‐2 infection.

To better understand the host responses to SARS‐CoV‐2 infection, gene ontology (GO) enrichment analysis was performed to identify biological processes enriched among significantly regulated genes. It appeared the distinctive GO terms were enriched between HPAEpiC cells and HULEC‐5a cells following viral infection. Notably, among the top 20 enriched terms, the biological processes associated with epithelial cell apoptosis, cell division, and mitotic cell cycle were particularly enriched in HPAEpiC cells (Figure 4E). While, in HULEC‐5a cells, the biological processes involved in cytokinesis, transcriptional factor activity, and chemotaxis were significantly modulated (Figure 4F).

**Figure 5:**
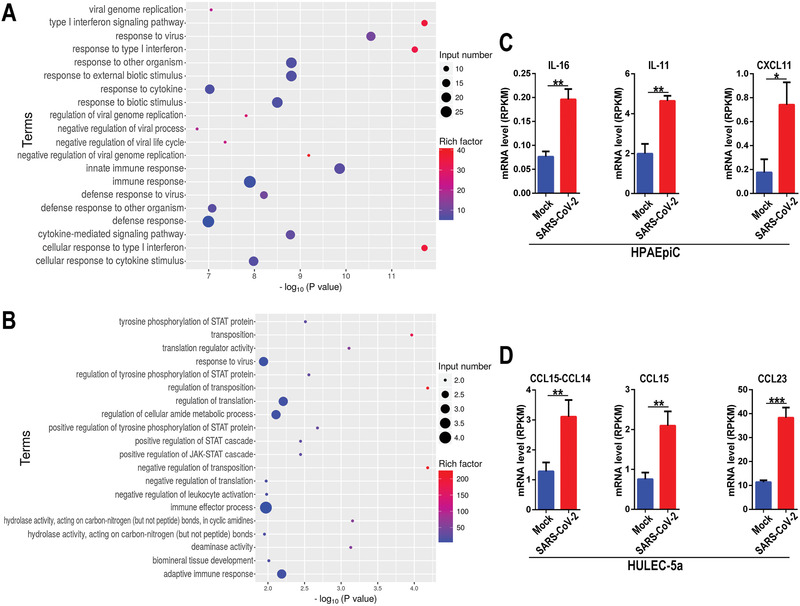
RNA‐seq analysis showed distinctive immune responses to SARS‐CoV‐2 infection in cocultured HPAEpiC cells and HULEC‐5a cells on chip. A) GO enrichment analysis of upregulated DEGs related with immune response in HPAEpiC cells after viral infection. F) GO enrichment analysis upregulated DEGs related with immune response in HULEC‐5a cells after viral infection. C) Upregulated cytokines genes in HPAEpiC cells infected with virus by RNA‐seq analysis. D) Upregulated cytokines genes in HULEC‐5a cells infected with virus by RNA‐seq analysis. Data were analyzed by Student's *t*‐test (*p < 0.05).

In general, viral infection can trigger antiviral or immune responses in host cells. In order to identify the specific host defense responses to SARS‐CoV‐2 infection, we then searched the genes related with immune responses from the upregulated DEGs according to GO annotation, and performed GO enrichment analysis. The results demonstrated distinct immune responses induced by this virus in the two cell types. It is noted, SARS‐CoV‐2 infection induced a broad innate immune response and antiviral responses, including defense response to virus, type I interferon (IFN‐I) signaling pathway, and cytokine‐mediated signaling pathway in HPAEpiC cells (**Figure** **5**A). While, the terms related to the positive regulation of JAK‐STAT cascade and adaptive immune response were particularly enriched in HULEC‐5a cells (Figure 5B). Moreover, among the upregulated genes associated with immune responses, we identified some cytokines, including IL‐16, IL‐11, and CXCL11 in HPAEpiC cells (Figure 5C), and CCL15‐CCL14, CCL15, and CCL23 in HULEC‐5a (Figure 5D).

Collectively, these findings revealed SARS‐CoV‐2 infection exhibited distinct effects on alveolar epithelial cells and microvascular endothelial cells, including the susceptibility to virus, immune responses, and activated signaling pathways. These data reflect the diverse responses of human alveolar epithelial and endothelial cells to viral infection, which may explain the possible complex cross‐talk of host cells involved in the pathogenesis of COVID‐19.

### 2.3 Immune Responses in Human Alveolus Chip Following SARS‐CoV‐2 Infection

**Figure 6:**
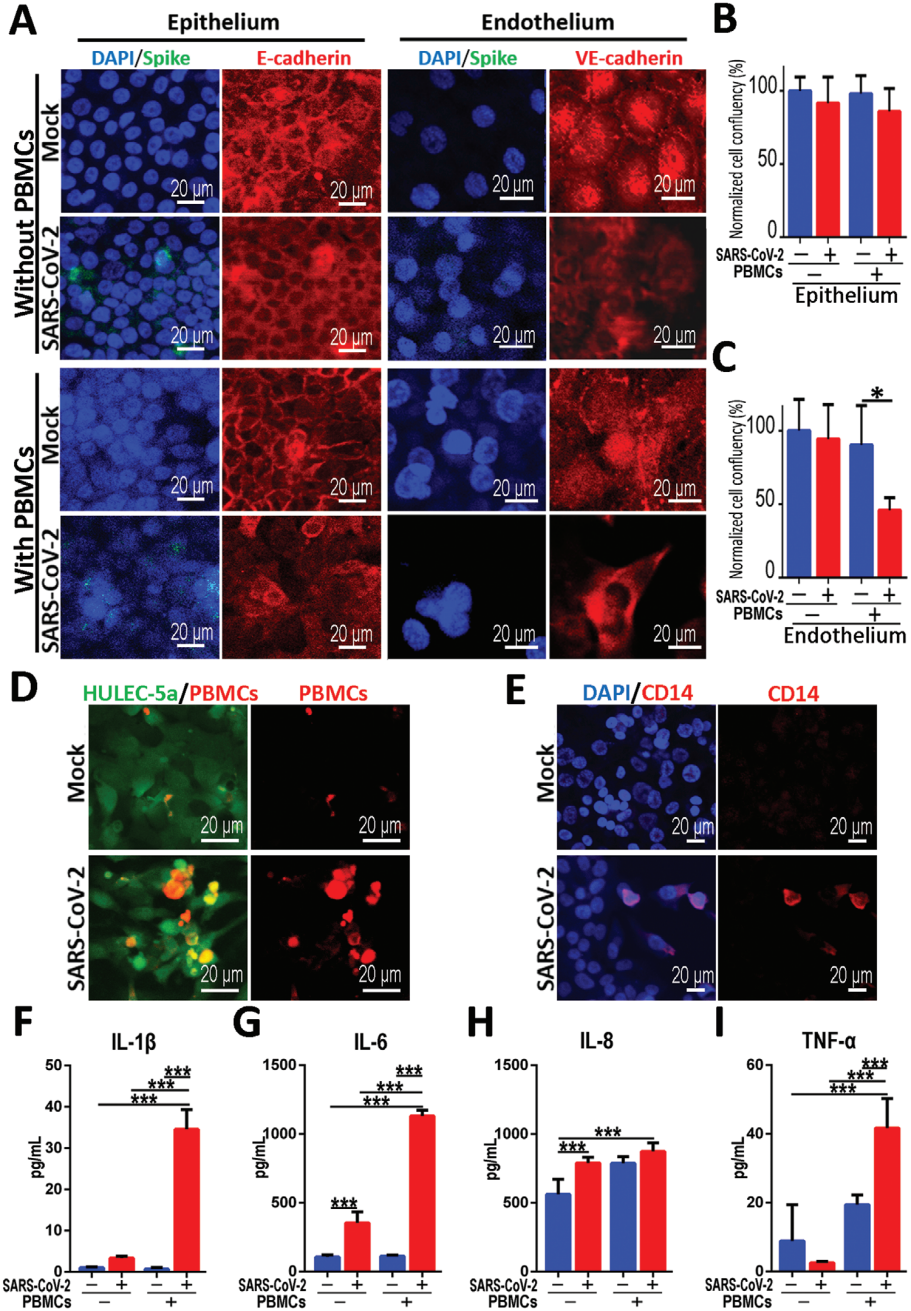
Distinctive responses of cocultured epithelium and endothelium to SARS‐CoV‐2 infection in the presence or absence of circulating immune cells. A) Confocal images showed the effects of SARS‐CoV‐2 infection (Spike) on the epithelium (E‐cadherin) and endothelium (VE‐cadherin) with or without immune cells (PBMCs) at day 2 post‐infection. B,C) Quantifications of cell confluency following SARS‐CoV‐2 infection in the presence or absence of circulating immune cells (PBMCs). Three chips were quantified for each group. Data were analyzed using a one‐way ANOVA with Bonferroni post‐test (*p < 0.05). D) Confocal immunofluorescent microscopy images showed the recruitment and adhesion of PBMCs (red) on the surface of HULEC‐5a layer (green) after SARS‐CoV‐2 infection. E) Confocal immunofluorescent microscopy images showed the recruitment and adhesion of CD14+ monocytes (red) on the surface of HULEC‐5a cell layer (green) after SARS‐CoV‐2 infection. F–I) Quantitative analysis of released inflammation cytokines IL‐1*β*, IL‐6, IL‐8, and TNF‐*α* from the culture medium in vascular channel under different conditions. Data were presented as mean ± SD. Data were analyzed using a one‐way ANOVA with Bonferroni post‐test (***p < 0.001). Six chips were quantified for each group.

The accumulation and extensive infiltration of immune cells in the lungs may contribute significantly to the pathogenesis in patients infected with respiratory viruses.^[^ ^38^ ^]^ We next explored the roles of human circulating immune cells in the alveolar pathological process after SARS‐CoV‐2 infection. In this study, PBMCs were isolated from healthy human blood and infused into the lower vascular channel of chip. SARS‐CoV‐2 was then inoculated into the upper channel at a MOI of 10 and incubated for 2 d. The infection of SARS‐CoV‐2 was identified in alveolar epithelial cells by Spike protein in the presence of PBMCs (**Figure** **6**A). Strikingly, in the presence of PBMCs, the distributions of junction proteins E‐cadherin and VE‐cadherin in human epithelial and endothelial cells were intensely disrupted after virus infection (Figure 6A). These results suggest the damage of the alveolar barrier integrity induced by viral infection in the presence of circulating immune cells. Furthermore, the cell confluency of human endothelium and epithelium after SARS‐CoV‐2 infection was examined by adding PBMCs in vascular channel. Notably, PBMCs caused a significant decrease of cell confluency in endothelial cells from 90.31 ± 26.91% to 45.92 ± 8.52% (Figure 6B,C), however, no significant changes of cell confluency were observed in epithelial cells. These data indicate the occurrence of cellular detachment and injury of vascular endothelial cells in the presence of circulating immune cells in this microsystem. It also reveals the crucial role of immune cells in mediating the dysfunction of alveolar barrier after SARS‐CoV‐2 infection.

According to GO enrichment analysis, terms of positive regulation of immunocytes chemotaxis, such as macrophage chemotaxis were particularly enriched in the infected endothelial cells. Subsequently, we examined the changes of circulating immunocytes in the vascular side of the chip after viral infection. Strikingly, the recruitment and adhesion of PBMCs, especially CD14^+^ monocytes (the precursor cells of a macrophage) were observed on the endothelium layer (Figure 6D,E), which was similar to the inflammatory cell infiltration in lungs as observed clinically.^[^ ^9^, ^39^ ^]^ In addition, we also applied the nonspecific stimulants (e.g., IL‐2 and IL‐6) on the alveolar chip to identify the response of human immune cells to these stimulators. The immune cells were introduced into the vascular channel of the chip after IL‐2 or IL‐6 (30 ng mL^−1^) treatment for 2 d. More immune cells were observed to be attached to the pulmonary microvascular endothelium induced by IL‐6, which is similar to the recruitment of immune cells in the vascular side induced by SARS‐CoV‐2 infection in our chip model. These results suggest that inflammatory factors (e.g., IL‐6) may play a crucial role in the activated immune response of host cells to viral infection (Figure S5, Supporting Information).

To further explore the inflammatory responses induced by SARS‐CoV‐2 in this model system, we evaluated the release of several pro‐inflammatory cytokines from culture media in the vascular channel. At day 2 post‐infection, the levels of IL‐6 and IL‐8 were significantly increased in the absence of PBMCs (Figure 6G,H). Moreover, all four cytokines (IL‐1*β*, IL‐6, IL‐8, and TNF‐*α*) were markedly increased following SARS‐CoV‐2 infection with the incorporation of PBMCs (Figure 6F–I). Especially, viral infection caused a tenfold increase of IL‐1*β* and IL‐6 level in the culture medium from the lower endothelium layer as compared to the group without infection (Figure 6F,G). Similarly, the increased secretion of these inflammatory cytokines from upper epithelium layer was also detected (Figure S6, Supporting Information). Clearly, SARS‐CoV‐2 infection in this alveolar chip could induce the recruitment of PBMCs, and aggravated inflammatory response in lung tissue as observed in clinics, demonstrating the feasibility of this microengineered lung model system to reflect the human‐relevant pathophysiology of COVID‐19.

### 2.4 Assessment of Potential Antiviral Therapeutics

**Figure 7:**
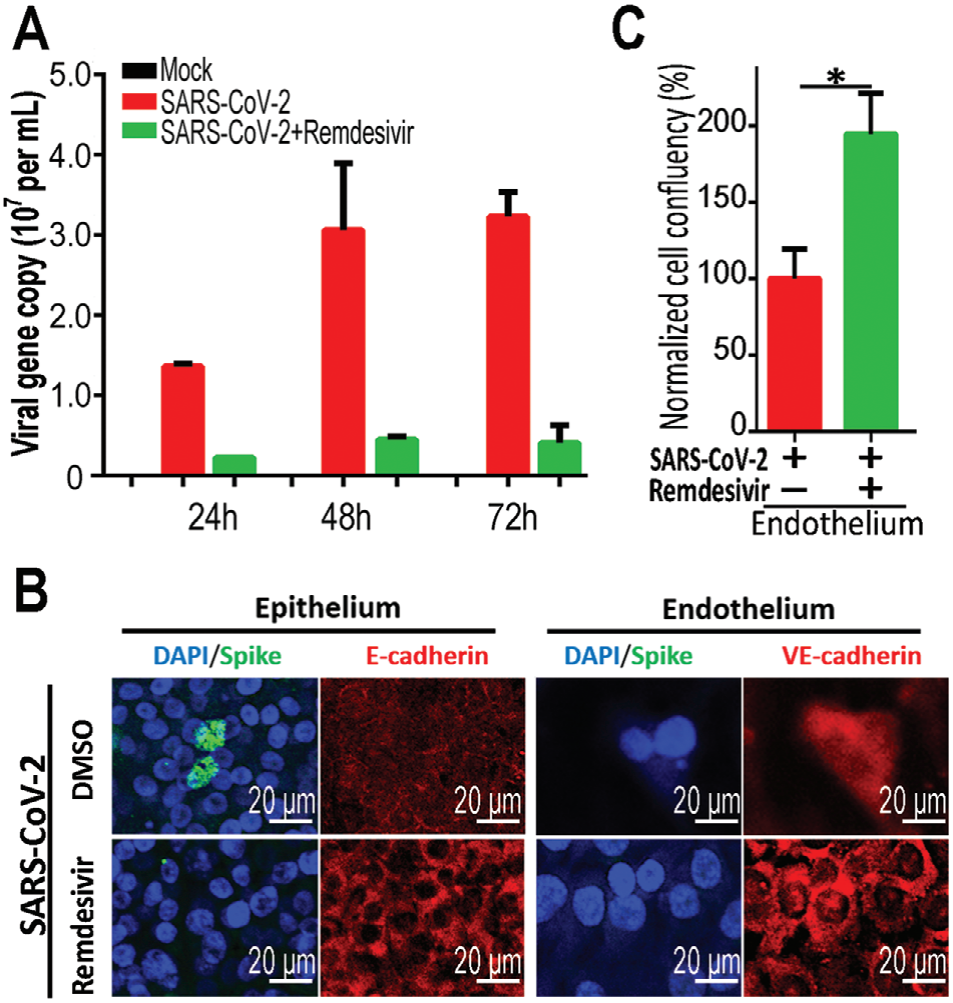
Evaluation of potential antiviral efficacy of remdesivir on the chip system. A) Culture supernatants were harvested at indicated time points following SARS‐CoV‐2 infection to examine the viral load using qRT‐PCR for different groups. The average of two independent experiments is shown. Data were presented as mean ± SD. B) Confocal immunofluorescent microscopy images of epithelium (E‐cadherin) and endothelium (VE‐cadherin) of alveolus chip treated without or with 1 × 10^−6^ m remdesivir at day 2 post‐infection. C) Quantification for epithelial cell confluency of alveolus chip treated without or with 1 × 10^−6^ m remdesivir at day 2 post‐infection. Three chips were quantified for each group. Data were analyzed by Student's *t*‐test (*p < 0.05).

To assess the potential therapeutics against SARS‐CoV‐2, we treated the virus‐infected human alveolus chip with remdesivir. Remdesivir is recognized as a promising antiviral compound against many RNA viruses (e.g., SARS, MERS‐CoV), including SARS‐CoV‐2.^[^ ^40^, ^41^, ^42^ ^]^ In this study, an indicated dose of remdesivir (1 × 10^−6^ m) was added into the monolayer culture of HPAEpiC cells at 1 h post‐infection of SARS‐CoV‐2. After administration for 3 d, the culture supernatants were collected for virus titers determination by real‐time quantitative PCR (qRT‐PCR). A marked decrease of virus titers was detected in the infected‐HPAEpiC cells following remdesivir treatment (**Figure** **7**A). Furthermore, we tested the antiviral efficacy of remdesivir in the infected chip model with the addition of PBMCs in the vascular channel. In contrast with Figure 6B,C, remdesivir treatment could restore the damage of epithelial layers and endothelial layer to some extent (Figure 7B,C). These results indicated the potential role of remdesivir in suppressing SARS‐CoV‐2 replication and alleviating the virus‐induced injury of alveolar‐capillary barrier. In later work, this alveolar infection model may also be used to explore the effects of remdesivir administration in combination with other repurposing drugs as therapeutics against COVID‐19.

## 3 Discussion

**Figure 8:**
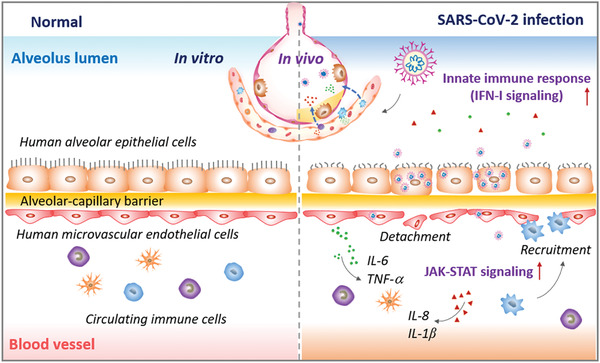
Schematic summary of SARS‐CoV‐2‐induced lung injury and inflammatory responses associated with COVID 19 on the human organ chip model system. Following SARS‐CoV‐2 exposure, virus particles invade the alveolar epithelium and endothelium and replicate violently in human epithelial cells. The viral infection can activate the host antiviral defense or immune response, including the activation of innate immune responses (e.g., IFN‐I signaling pathway) in epithelial cells and JAK‐STAT signaling pathway in endothelial cells. The cytokines or chemokines released from infected cells can recruit circulating immune cells (such as CD14^+^ monocytes) to infected sites and initiate inflammatory responses. This process further exacerbates the disruption of alveolus‐capillary barrier integrity, leading to lung injury.

In this study, we created a human disease model of SARS‐CoV‐2 infection on chip that can recapitulate the key pathophysiology and immune response of lung tissue associated with COVID‐19. The biomimetic human alveolus chip can resemble the alveolar‐capillary barrier injury and inflammatory response of lung after SARS‐CoV‐2 infection, such as viral replication in human alveolar epithelium, vascular dysfunction, recruitment of immune cells, and release of inflammatory cytokines in a physiological‐relevant manner (**Figure** **8**). In particular, we found circulating immune cells may largely contribute to the exacerbation of inflammatory responses and the injury of alveolar‐capillary barrier induced by SARS‐CoV‐2. These findings provide new insights into the pathogenesis of SARS‐CoV‐2, in which virus‐induced lung injury may be mediated by the complex and intrinsic cross‐talk among epithelium–endothelium interface and immune cells.

As we know, alveolar epithelial type II cells have been proved to be the primary target of SARS‐CoV‐2 infection by histopathological studies.^[^ ^43^ ^]^ In this study, we found that human alveolar epithelial cells are more susceptible to SARS‐CoV‐2 infection than endothelial cells identified on the chip. Moreover, transcriptomic analysis demonstrated the distinctive responses of alveolar epithelial and endothelial cells to SARS‐CoV‐2 infection, in which epithelium exhibited much higher viral load than that of endothelium. This may partially explain why SARS‐CoV‐2 cannot be easily detected in blood samples of COVID‐19 patients.^[^ ^37^ ^]^ In addition, compared with pulmonary microvascular endothelial cells, alveolar epithelial cells display a broader innate immune response after viral infection, such as IFN‐I signaling pathway. A recent study reported the IFN‐I responsive gene sets were upregulated in lung tissues from severe COVID‐19 cases, which may be associated with exacerbated lung inflammation.^[^ ^44^ ^]^ Our results are similar to these clinical findings, indicating the advantage of this disease model to reflect the human‐relevant immune responses of lung. Meanwhile, we detected the activation of JAK‐STAT signaling pathway in the infected pulmonary microvascular endothelial cells as compared to noninfected cells. It has been recognized that cytokines can activate JAK‐STAT pathway and regulate different cellular and immune processes.^[^ ^45^, ^46^ ^]^ Clinical studies reported Ruxolitinib, a JAK inhibitor, can effectively relieve the symptoms of patients with severe COVID‐19.^[^ ^47^, ^48^ ^]^ In combination with these findings, it might suggest the feasibility to develop new therapeutics against SARS‐CoV‐2 by targeting JAK‐STAT signaling pathway in microvascular endothelium.

Clinically, severe COVID‐19 patients often show inflammatory cytokine storms, which are associated with excessive immune responses, and may aggravate respiratory failure and cause multi‐organ damage. Circulating cytokines, including IL‐1*β*, IL‐6, IL‐8, and TNF‐*α* were significantly elevated in patients with severe COVID‐19.^[^ ^49^, ^50^ ^]^ As such, we compared the secretion of these cytokines in this alveolus chip under different treatment conditions. Our results showed that SARS‐CoV‐2 infection triggered the increased secretions of cytokines IL‐6 and IL‐8 on the alveolus chip. In particular, the addition of circulating immune cells caused the increased level of all these cytokines secretion, accompanied by recruitment of immune cells, detachment of endothelial cells, and severe disruption of intercellular junctions in both epithelium and endothelium. These findings are highly relevant with the pathological manifestations observed in severe COVID‐19 patients clinically,^[^ ^51^, ^52^ ^]^ verifying the role of immune cells in mediating alveolar injury, microvascular endothelial dysfunction, and excessive inflammatory response. Notably, the microvascular endothelial injury might explain the pathogenesis of microvascular thrombosis existed in the lung of severe COVID‐19 patients.^[^ ^53^, ^54^ ^]^

This work still has some limitations. In vivo, human lung alveolus consists of multiple cell types of pneumocytes (e.g., alveolar epithelial type I and type II cells). Here, we used only one cell type of alveolus epithelium, which may not fully simulate native alveolar tissues. The primary alveolar tissue from human biopsy can be selected for further studies. In addition, we use this model to assess only one type of antiviral candidates, remdesivir. Actually, the advantages and capabilities of this chip model system make it possible to evaluate other candidate antiviral agents, inflammatory cytokine inhibitors, or repurposing potential drugs against COVID‐19 in future. Due to the limited time and challenging conditions, in this work, we are still not able to conduct very deep research of SARS‐CoV‐2 pathogenesis or drug pharmacology. Nevertheless, the great value of this disease model on chip is that it is capable to reflect the human‐relevant pathophysiology of human lung and host‐immune response to this novel virus at organ level, which is difficult to be obtained by existing cell‐based system. This bioengineered lung infection model could provide a complement to animal models to evaluate drug candidates and repurpose approved drugs to face the crisis of SARS‐CoV‐2 epidemic.

## 4 Conclusions

Conclusively, this work made the first attempt to build a human alveolar infection model by SARS‐CoV‐2 using organ chip that allows to recapitulate the lung injury and immune response to viral infection in vitro at organ level. This model reflects the human‐relevant responses of alveolar‐capillary barrier to viral infection by integrating multicellular components, tissue–tissue interface, fluid flow, and circulating immune cells, thus narrowing the gap between in vitro models and human organ pathophysiology. The transcriptional analysis exhibited the activated innate immune responses in epithelium and activated cytokine‐dependent pathways in endothelium induced by SARS‐CoV‐2, which provides new insights into understanding the pathogenesis associated with COVID‐19. The recruitment of circulating immune cells is a major contributor to exacerbating lung inflammation, endothelial dysfunction, and injury of lung barrier caused by SARS‐CoV‐2, revealing the possible complex cross‐talk between the epithelium–endothelium and immune cells involved in the progression of COVID‐19.

This work provides the proof‐of‐concept to create a human disease model on chip to study host–virus interactions and human‐relevant responses at organ level. The obvious advantages of this chip platform lie in its accessibility to study responses of various cells to virus in real time simultaneously, and to rapidly test candidate drugs with low cost and short time, which cannot be easily achieved by existing in vitro experimental models or animal models. Moreover, it provides a synthetic strategy with flexibility for incorporating varying elements, thus can be adapted to emerging needs in the case of the current COVID‐19 pandemic.

## 5 Experimental Section

##### Device Fabrication

The human alveolus chip device consists of an upper and lower layer fabricated by casting PDMS prepolymer on molds prepared using conventional soft lithography procedures. 10:1 (wt/wt) PDMS base to curing agent (184 Silicone Elastomer, Dow Corning Corp) was polymerized to produce molded device with channels by thermal curing at 80 °C for 45 min. The top channel (1.5 mm wide × 0.25 mm high) and bottom channel (1.5 mm wide × 0.25 mm high) were used to form the alveolus lumen and the microvascular layer, respectively. The length of the overlapping channels is 15 mm. The two channels are separated by a thin (≈25 µm) through‐hole PDMS membrane (5 µm pores in diameter and 70 µm gaps between pores) to construct the tissue–tissue interface (Figure S1, Supporting Information). The porous PDMS membranes were fabricated based on the glass templates and spin‐coating method modified from the previous protocol. The membrane was sandwiched between the aligned upper and bottom channels of the device by oxygen plasma bonding for 30 s. Finally, the chip devices were sterilized in an autoclave and the porous member of the device was coated with rat tail collagen type I (200 µg mL^−1^, Corning) on both sides at 37 °C for 48 h before cell seeding.

##### Cell Culture and Lab Consumables

African green monkey kidney epithelial Vero E6 cells (ATCC, no. 1586) were cultured in Dulbecco's modified Eagle's medium (DMEM, Gibco) supplemented with 10% fetal bovine serum (FBS, Gibco). Immortalized human alveolar epithelial cells (HPAEpiC) were generated from Type II pneumocytes of human lung tissue (purchased form Sciencell Shanghai Corporation) and were maintained in RPMI 1640 medium (Gibco) supplemented with 10% FBS. Human lung microvasculature cell line HULEC‐5a was purchased from Procell Corporation and were maintained in HULEC‐5a growth medium (Procell, CM‐0565). Human peripheral blood mononuclear cells were isolated from fresh human blood using Ficoll (Stem cell technologies) density centrifugation. Isolated PBMCs were resuspended in RPMI 1640 medium containing 10% FBS and 50 IU mL^−1^ IL‐2 and used for adhesion assays on chip. All cells were cultured at 37 °C in a humidified atmosphere of 5% CO_2_.

To create the human alveolus chip, HULEC‐5a cells (≈1 × 10^5^ cells) were initially seeded on the bottom side of the collagen‐coated porous PDMS membrane and then attached to the membrane surface under static conditions for 2 h. Subsequently, cells were washed with fresh medium to exclude/remove unattached ones. Then, HPAEpiC cells (≈5 × 10^5^ cells) were seeded into the upper channel under static culture. After cell attachment, constant media flows (50 µL h^−1^) were applied in both the upper and bottom layers using peristaltic pump. The cells were grown to confluence for 3 d and the chips were maintained in an incubator with 5% CO_2_ at 37 °C. All lab consumables were bought from Guangzhou Jet Bio‐Filtration Co., Ltd.

##### Virus

The clinical isolate SARS‐COV‐2 strain 107 was obtained from Guangdong Provincial Center for Disease Control and Prevention, China, and propagated in Vero E6 cells. The virus titer (the infectious titer of virus) was determined by a TCID50 assay on Vero cells. The MOI was calculated according to the formula: MOI = TCID50 × 0.7/cell number. All work involving live SARS‐CoV‐2 was performed in the Chinese Center for Disease Control and Prevention‐approved BSL‐3 laboratory of the Kunming Institute of Zoology, Chinese Academy of Science.

##### SARS‐CoV‐2 Infections

HPAEpiC cells were seeded in 24‐well plates (2 × 10^5^ cells per well) in RPMI 1640 medium containing 10% FBS. After seeding for 24 h, cells were infected with SARS‐CoV‐2 at a MOI of 10. After 1 h, cells were washed three times with phosphate buffered saline (PBS) and kept in fresh medium for 3 d. At 3 d postinfection, cells were washed with PBS and then fixed with 4% paraformaldehyde (PFA) before analysis. The culture supernatant was collected for RNA extraction.

For SARS‐CoV‐2 infection in the human alveolus chip, the apical channel of chip device was infused with 30 µL of RPMI 1640 medium containing the indicated multiplicity of virus (MOI = 10). After 1 h of infection, cells were washed three times with PBS and kept in fresh medium. At day 3 post‐infection, the apical and basal media were collected for analysis of released cytokines. The HPAEpiC and HULEC‐5a cells cultured on the chip were fixed for immunofluorescence analysis or lysed for RNA‐seq data analysis, respectively.

##### Permeability Assay

The alveolar‐capillary barrier permeability was assessed by detecting the diffusion rate of FITC‐dextran from the lower vascular layer to the upper alveolar channel. After 3 d of cocultivation, the medium with FITC‐dextran (40 kDa, 1 mg mL^−1^) was then infused into the bottom channel of the device. The media were collected from the upper channel at different time points (0, 1, and 2 h) and the fluorescence intensity was measured using microplate system (ABI Vii 7).

##### Western Blot Analysis

The protein samples were separated on 10% sodium dodecyl sulfate polyacrylamide gel electrophoresis (SDS‐PAGE) and then transferred onto 0.2 µm nitrocellulose membranes (GE Amersham). After being blocked with 5% bovine serum albumin (BSA) in Tris Buffered saline with 0.05% Tween (TBST buffer) containing 0.05% Tween‐20, the membranes were probed with primary antibodies at 4 °C overnight. The membranes were then probed with horseradish peroxidase (HRP)‐conjugated secondary antibodies for 1 h at room temperature. Protein bands were detected by Prime Western Blotting Detection Reagent (GE life).

##### Immunostaining

HPAEpiC cells cultured on well plate were washed with PBS and fixed with 4% PFA at 4 °C overnight. Cells were then permeabilized with 0.2% Triton X‐100 in PBS (PBST buffer) for 5 min and blocked with PBST buffer containing 5% normal goat serum for 30 min at room temperature. Antibodies were diluted with PBST buffer. Cells were stained with corresponding primary antibodies at 4 °C overnight and with secondary antibodies (Table S1, Supporting Information) at room temperature for 1 h. After staining with the secondary antibodies, the cell nuclei were counterstained with 4′,6‐diamidino‐2‐phenylindole (DAPI).

For immunofluorescent imaging of alveolus chip, cells were washed with PBS through the upper and bottom channels and fixed with 4% PFA. The fixed cells were subjected to immunofluorescence staining by the same procedure as described above. All images were acquired using a confocal fluorescent microscope system (Carl Zeiss LSM880). Image processing was done using ImageJ (NIH).

##### Analysis of Inflammatory Cytokines

To analyze the released cytokines, the media in the alveolar channel and vessel channel were collected from each chip, respectively. The concentrations of IL‐6, IL‐8, IL‐1*β*, and TNF‐*α* were measured using the corresponding human ELISA kits (Biolegend, USA) according to the manufacturer's instructions.

##### Real‐Time Quantitative PCR

Virus titers were determined by viral RNA detection using qRT‐PCR. The culture supernatant for each condition was harvested for RNA extraction using the HP Viral RNA Kit (Roche, Cat No. 11858882001) according to the manufacturer's instructions. QRT‐PCR was performed using One Step RT‐PCR RNA direct real‐time PCR master mix (TOYOBO, QRT‐101A) in a PCR System (Applied Biosystem, ViiA 7). The primers of SARS‐CoV‐2 E gene were as follows: N‐F: 5′‐GGG GAA CTT CTC CTG CTA GAA T‐3′; N‐R: 5′‐CAG ACA TTT TGC TCT CAA GCT G‐3′; N‐probe: 5′‐TTG CTG CTG CTT GAC AGA TT‐3′. PCR amplification was performed under the following conditions: 50 °C for 10 min, and 95 °C 1 min, followed by 45 cycles consisting of 95 °C for 15 s, 60 °C for 45 s.

##### Transmission Electron Microscopy

HPAEpiC cells were collected and fixed in 4% PFA (Electron Microscopy Sciences) and 2.5% glutaraldehyde (Electron Microscopy Sciences) at 4 °C overnight. After washed with PBS for three times and fixed in 1% OsO_4_ buffer for 2 h, the samples were dehydrated with graded ethanol solutions, and embedded in Epon 812 resin (SPI). Ultrathin sections (70 nm) were stained with 2% uranyl acetate for 30 min and then lead citrate for 10 min. Images were acquired with a JEM‐1400 PLUS electron microscope.

##### Remdesivir Treatment

Stock solution of remdesivir (GS‐5734) was prepared in dimethyl sulfoxide (DMSO). For the HPAEpiC cell culture on the plate, after SARS‐CoV‐2 infection for 1 h, the cells were treated with or without 1 × 10^−6^ m remdesivir for 3 d and the supernatants were collected at distinct time points (24, 48, and 72 h) for viral load analysis by qRT‐PCR. For alveolus chip, cells were treated with or without 1 × 10^−6^ m remdesivir for 2 d after SARS‐CoV‐2 infection in the presence of PBMCs. After 48 h, the cells were fixed for immunofluorescence analysis.

##### RNA Extraction, Library Preparation, and Sequencing

HPAEpiC cells and HULEC‐5a cells were collected separately from the chips, and total RNAs were extracted from samples using TRIzol (Invitrogen) following the methods by Chomczynski and Sacchi.^[^ ^55^ ^]^ DNA digestion was carried out after RNA extraction by DNaseI. RNA quality was determined by examining A260/A280 with Nanodrop OneCspectrophotometer (Thermo Fisher Scientific Inc.). RNA integrity was confirmed by 1.5% agarose gel electrophoresis. Qualified RNAs were finally quantified by Qubit 3.0 with Qubit RNA Broad Range Assay kit (Life Technologies). 500 ng total RNAs were used for stranded RNA sequencing library preparation using KC‐Digital Stranded mRNA Library Prep Kit for Illumina (Catalog No. DR08502, Wuhan SeqHealth Co., Ltd., China) following the manufacturer's instruction. The kit eliminates duplication bias in PCR and sequencing steps, using unique molecular identifier (UMI) of eight random bases to label the preamplified cDNA molecules. The library products corresponding to 200–500 bps were enriched, quantified, and finally sequenced on Hiseq X 10 sequencer (Illumina).

##### RNA‐seq Data Analysis

Raw sequencing data was first filtered by Trimmomatic (version 0.36), low‐quality reads were discarded, and the reads contaminated with adaptor sequences were trimmed. Clean reads were further treated with in‐house scripts to eliminate duplication bias introduced in library preparation and sequencing. In brief, clean reads were first clustered according to the UMI sequences, in which reads with the same UMI sequence were grouped into the same cluster, resulting in 65 536 clusters. Reads in the same cluster were compared to each other by pairwise alignment, and then reads with sequence identity over 95% were extracted to a new subcluster. After all subclusters were generated, multiple sequence alignment was performed to get one consensus sequence for each subcluster. After these steps, any errors and biases introduced by PCR amplification or sequencing were eliminated.

The de‐duplicated consensus sequences were used for standard RNA‐seq analysis. They were mapped to the reference genome of *Homo sapiens* from Ensembl database (ftp://ftp.ensembl.org/pub/release-87/fasta/homo_sapiens/dna/) using STAR software (version 2.5.3a) with default parameters. Reads mapped to the exon regions of each gene were counted by featureCounts (Subread‐1.5.1; Bioconductor) and then RPKMs were calculated. Differentially expressed genes between groups were identified using the edgeR package (version 3.12.1). An FDR‐corrected *p*‐value cutoff of 0.05 and fold‐change cutoff of 2 were used to judge the statistical significance of gene expression differences. GO enrichment analysis for differentially expressed genes was implemented by KOBAS software (version: 2.1.1) with a corrected *P*‐value cutoff of 0.05 to judge whether it has statistically significant enrichment.

##### Statistical Analyses

Data were collected in Excel (Microsoft). The difference between two groups was analyzed using Student's *t*‐test. Multiple group comparison was performed using one‐way analysis of variance (ANOVA) followed by Bonferroni post hoc test. The bar graphs with error bars represent mean ± standard deviation (SD). Significance is indicated by asterisks: *p < 0.05; **p < 0.01; ***p < 0.001.

## Conflict of Interest

The authors declare no conflict of interest.

## Supporting information

Supporting Information

## Data Availability

All relevant data are available in the manuscript or Supporting Information. All of the RNA‐seq raw data have been deposited on SRA under the accession number PRJNA647532.
