# Supplementary material for: Biomimetic Human Disease Model of SARS‐CoV‐2‐Induced Lung Injury and Immune Responses on Organ Chip System
Source: Adv Sci (Weinh). 2020 Dec 21;8(3):2002928. doi: 10.1002/advs.202002928 (PMC7646023; doi:10.1002/advs.202002928)
Supplement: Supplementary file 1 — Supporting Information [file ADVS-8-2002928-s001.pdf]

© 2020 Wiley-VCH GmbH

ADVANCED SCIENCE
Open Access

Supporting Information

for *Adv. Sci.,* DOI: 10.1002/advs.202002928

Biomimetic human disease model of SARS-CoV-2 induced lung injury and immune responses on organ chip system

Min Zhang[1,4] †, Peng Wang[1] †, Rong-Hua Luo[5,6,7] †, Yaqing Wang[1,4] †, Zhongyu Li[1], Yaqiong Guo[1,4], Yu-Lin Yao[6,8], Ming-Hua Li[5], Tingting Tao[1,4], Wenwen Chen[1,4], Jian-Bao Han[5], Haitao Liu[1,4], Kangli Cui[1,4], Xu zhang[1], Yong-Tang Zheng[5,6,7], Jianhua Qin[1,2,3,4,*]*

Supplementary Information:

Biomimetic human disease model of SARS-CoV-2 induced lung injury and immune responses on organ chip system

Min Zhang[1,4] †, Peng Wang[1] †, Rong-Hua Luo[5,6,7] †, Yaqing Wang[1,4] †, Zhongyu Li[1], Yaqiong Guo[1,4], Yu-Lin Yao[6,8], Ming-Hua Li[5], Tingting Tao[1,4], Wenwen Chen[1,4], Jian-Bao Han[5], Haitao Liu[1,4], Kangli Cui[1,4], Xu zhang[1], Yong-Tang Zheng[5,6,7*], Jianhua Qin[1,2,3,4,*]

†These authors contributed equally to this work.

[1]Division of Biotechnology, CAS Key Laboratory of SSAC, Dalian Institute of Chemical Physics, Chinese Academy of Sciences, Dalian, China.

[2]Institute for Stem Cell and Regeneration, Chinese Academy of Sciences, Beijing, China

[3]CAS Center for Excellence in Brain Science and Intelligence Technology, Chinese Academy of Sciences, Shanghai, China

[4]University of Chinese Academy of Sciences, Beijing, China

[5]Kunming National High-level Bio-safety Research Center for Non-human Primates, Center for Biosafety Mega-Science, Kunming Institute of Zoology, Chinese Academy of Sciences, Kunming, 650107, China

[6]Key Laboratory of Animal Models and Human Disease Mechanisms of Chinese Academy of Sciences and Yunnan Province, Kunming Institute of Zoology, Chinese Academy of Sciences, Kunming, Yunnan 650223, China

[7]KIZ-CUHK Joint Laboratory of Bioresources and Molecular Research in Common Diseases, Kunming Institute of Zoology, Chinese Academy of Sciences, Kunming, Yunnan 650223, China.

[8]Kunming College of Life Science, University of Chinese Academy of Sciences, Kunming, Yunnan 650204, China

***Correspondence:**

Jianhua Qin, Division of Biotechnology, Dalian Institute of Chemical Physics, Chinese Academy of Sciences, 457 Zhongshan Road, Dalian 116023, China. E-mail: jhqin@dicp.ac.cn. Tel: 86-0411-84379650.

Yongtang Zheng, Kunming Institute of Zoology, 32 Jiaochang Donglu Kunming, Yunnan, E-mail: zhengyt@mail.kiz.ac.cn. Tel: +86 0871 65295684.

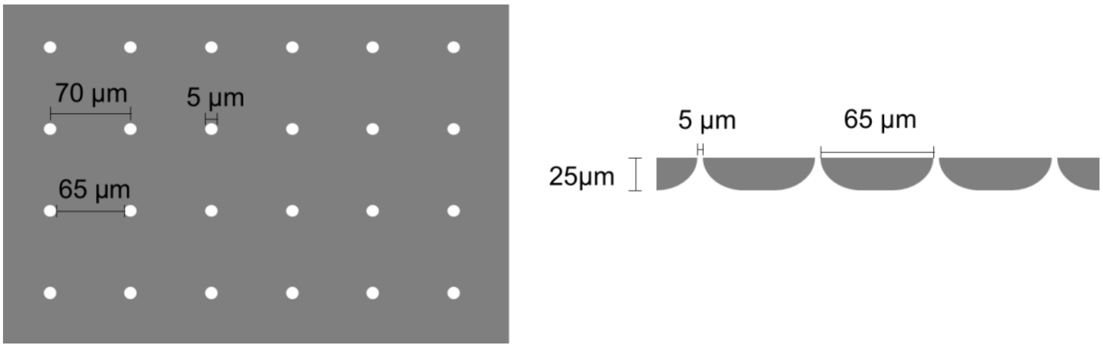

Figure S1. Schematic diagram of porous PDMS membrane used in alveolus chip. The diameter of the pore is 5 μm, and the center-to-center spacing between the two adjacent pores is 70 μm. The thickness of the membrane is 25 μm.

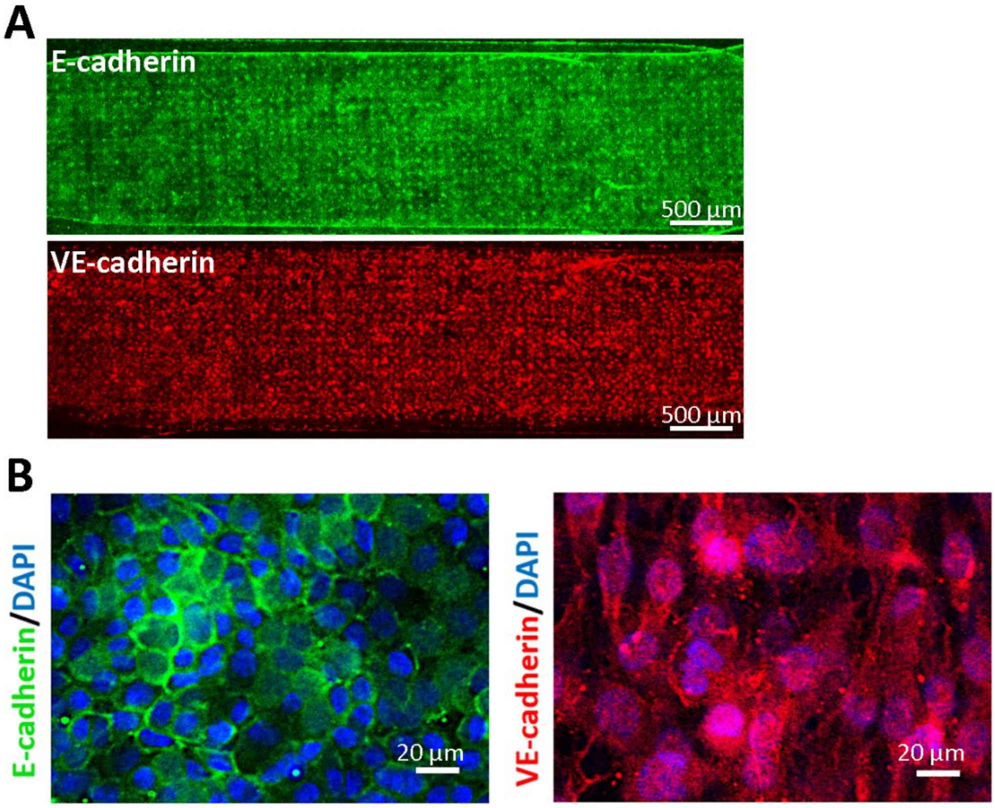

Figure S2. Characterization of the formed alveolar epithelium and pulmonary microvascular endothelium in the human alveolus chip by confocal immunofluorescent imaging. (A) The full scanning of epithelial layer (E-cadherin) and endothelial layer (VE-cadherin) in the parallel channel. (B) The formation of adherent junctions in alveolar layer (E-cadherin) and microvascular endothelium (VE-cadherin) on alveolus chip.

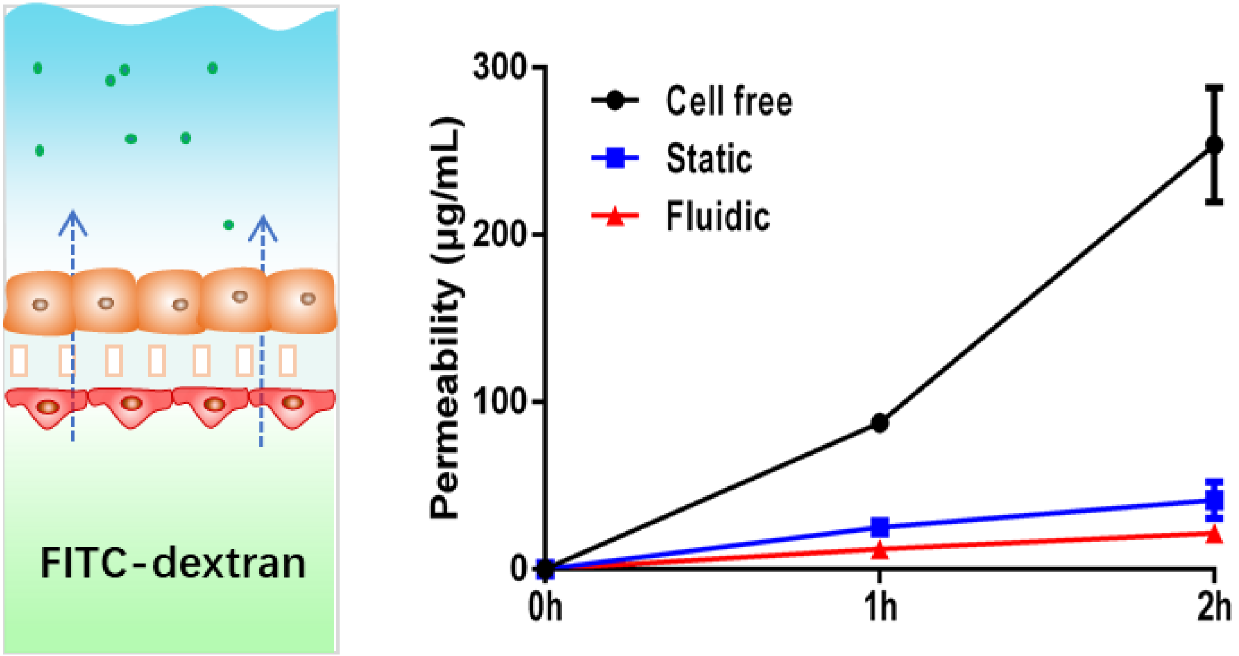

Figure S3. Permeability testing of the formed alveolar-capillary barrier within alveolus chip under different culture conditions. After cell loading for 3 days, the medium with FITC-dextran (40 kDa, 1 mg/mL) was infused into the vascular channel of device. Then, the media was collected from the alveolar channel at different time points (0, 1, 2 h) and the fluorescence intensity was measured to evaluate the permeability of the alveolar capillary barrier. Data were presented as mean ± SD. N=2.

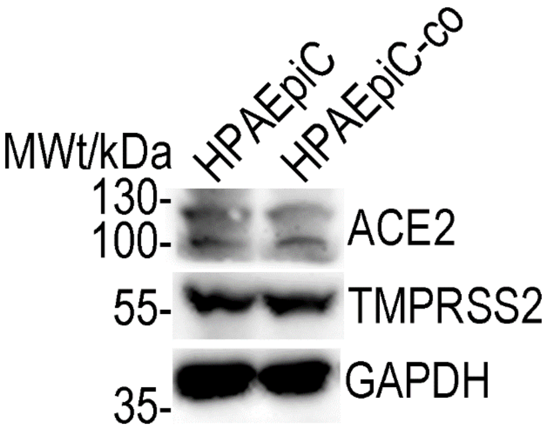

Figure S4. The expression levels of ACE2 and TMPRSS2 proteins in HPAEpiC cells were examined by western blot under mono-cultures or co-cultures with HULEC-5a cells in Transwell for 3 days.

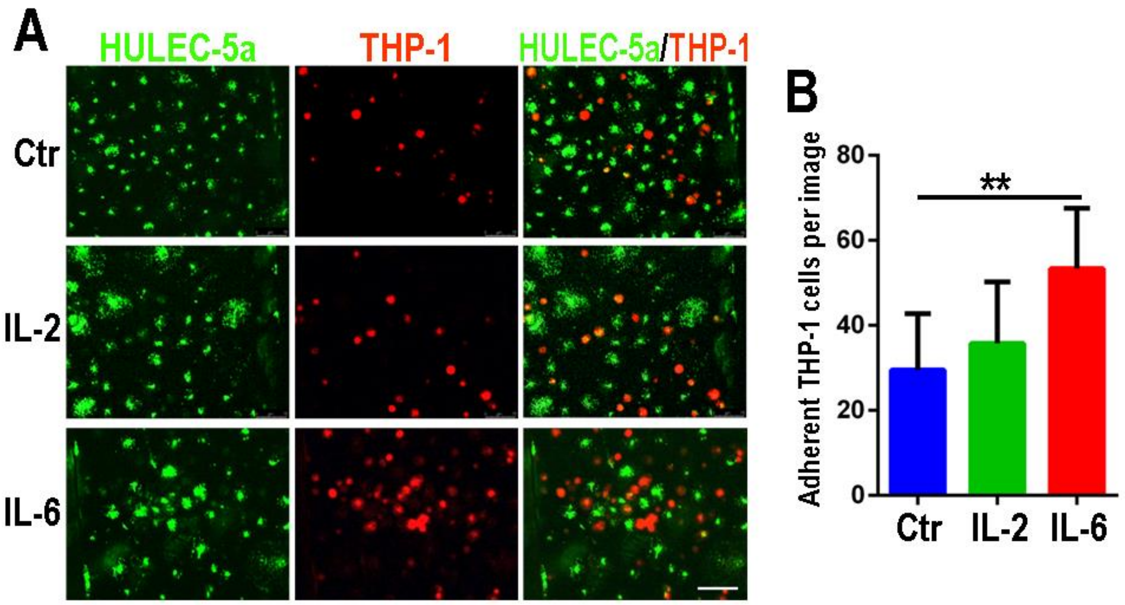

Figure S5. Confocal immunofluorescent images of THP-1 monocyte adhesion to HULEC-5a cells after exposure to IL-6 or IL-2 in the microfluidic chip. (A) The HULEC-5a cells were treated with 30 ng/mL IL-2 or 30 ng/mL IL-6 for two days. Then the THP-1 cells were introduced into the vascular channel of the chip. Before infusion into the vascular channel, THP-1 cells were activated with 10 ng/mL phorbol myristrate acetate (PMA). Confocal immunofluorescent micrographs showed the adhesion of THP-1 cells (Red) on the surface of HULEC-5a cell layer (Green). Scale bars: 100 μm. (B) Quantitative analysis of the number of adherent THP-1 cells per image in the control and groups treated with IL-2 and IL-6. Data were presented as mean ± SD. Data were analyzed using one-way ANOVA with Bonferroni post-test (***: $p<0.001$). Eight chips were quantified for each group.

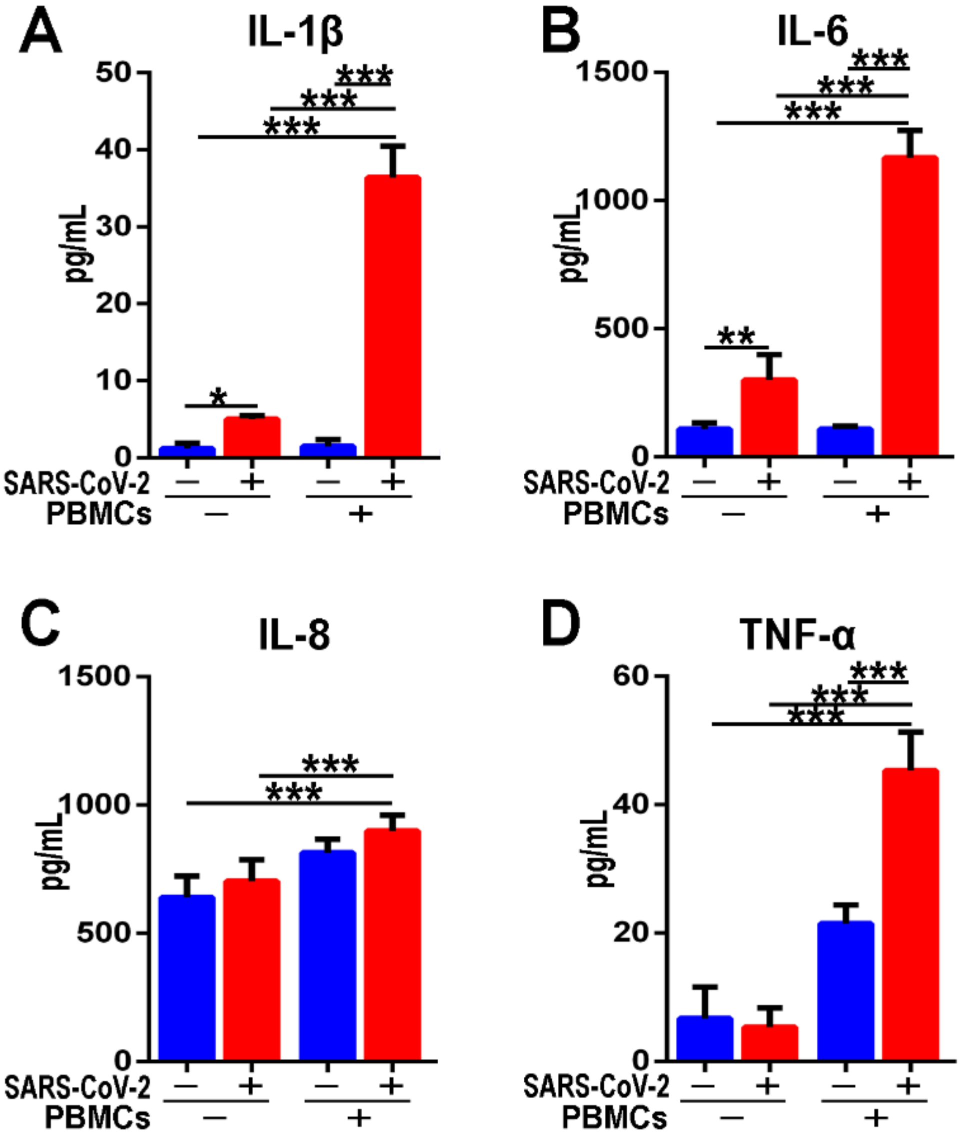

Figure S6. Examination of cytokines produced from epithelium effluence after SARS-CoV-2 infection in the presence or absence of PBMCs on the alveolar chip. Quantitative analysis of the secreted inflammatory cytokines (A) IL-1β, (B) IL-6, (C) IL-8, and (D) TNF-α in media from the alveolar layer. Data were presented as mean ± SD. Data were analyzed using a one-way ANOVA with Bonferroni post-test (***: $p<0.001$). Six chips were quantified for each group.

Table S1. List of antibodies used for Western blot (WB) and immunofluorescence (IF).

Primary antibodies	Supplier	Cat #	Species	Purpose	Dilution
ACE2	Proteintech Group	21115-1-AP	rabbit	WB	1:1000
TMPRSS2	Proteintech Group	14437-1-AP	rabbit	WB	1:1000
GAPDH	CWBIO	CW0100	rabbit	WB	1:5000
Nucleoprotein (NP)	SinoBiological	40143-R019-100	rabbit	WB	1:1000
E-caherin	Proteintech Group	60335-1-Ig	mouse	IF	1:200
VE-cadherin	Proteintech Group	66804-1-Ig	mouse	IF	1:200
CD14	Abcam	Ab183322	rabbit	IF	1:200
Spike	Sino Biological	40150-R007	rabbit	IF	1:200

Secondary antibodies	Supplier	Cat #	Dilution
Anti-Rabbit IgG(H+L), F(ab')2 Fragment (Alexa Fluor® 488 Conjugate)	Cell Signaling	4412	1:1000
Anti-Rabbit IgG(H+L), F(ab')2 Fragment (Alexa Fluor® 594 Conjugate)	Cell Signaling	8889	1:1000
Anti-mouse IgG (H+L), F(ab')2 Fragment (Alexa Fluor® 594 Conjugate)	Cell Signaling	8890	1:1000
Anti-mouse IgG (H+L), F(ab')2 Fragment (Alexa Fluor® 488 Conjugate)	Cell Signaling	4408	1:1000
